# Individual differences in hearing decline predict region-specific brain and cognitive changes in healthy aging men

**DOI:** 10.3389/fragi.2026.1867663

**Published:** 2026-07-29

**Authors:** Kamine Julie Jacobsen, Mostafa Mehdipour Ghazi, Reena Murmu Nielsen, Elaine Hoi Ning Ng, Nelly Shenton, Line Skødebjerg Kristensen, Olalla Urdanibia-Centelles, Sine Kongsbak Arvedsen, Henrik Bo Wiberg Larsson, Mark Bitch Vestergaard, Martin Lauritzen, Krisztina Benedek

**Affiliations:** 1 Department of Neurology, Section of Clinical Neurophysiology, Zealand University Hospital, Roskilde, Denmark; 2 Department of Computer Science, Pioneer Centre for AI, University of Copenhagen, Copenhagen Ø, Denmark; 3 Oticon A/S, Smørum, Denmark; 4 Department of Neurophysiology, Rigshospitalet, Glostrup, Denmark; 5 Faculty of Health and Medical Sciences, University of Copenhagen, Copenhagen, Denmark; 6 Department of Clinical Physiology and Nuclear Medicine, Rigshospitalet, Copenhagen, Denmark

**Keywords:** cognition, deep learning, healthy aging, MRI, presbiacusis

## Abstract

**Background:**

Cognitive decline in aging is heterogeneous and accelerates after approximately 60 years of age. Hearing decline is a common, potentially modifiable risk factor that may contribute to individual differences in cognitive aging and brain structural change. In addition to its cognitive consequences, hearing loss can impair communication and contribute to reduced social participation, loneliness, and poorer quality of life. However, the extent to which longitudinal changes in hearing predict cognitive performance and regional brain structure remains unclear.

**Methods:**

We assessed 51 healthy males from a longitudinal birth cohort extensively evaluated with cognitive testing and MRI across the lifespan. At ages 70 and 71 (recruited in 2024), participants completed four-frequency pure-tone audiometry (4PTA), audible contrast threshold (ACT), speech-in-noise tests (HINT), cognitive assessments, and structural MRI scans. All participants had a prior visit at age 63 ± 1 year (recruited in 2015–2018) with the same cognitive test battery. Previously, machine learning algorithm models were used to identify cognitive decline, and participants were clustered into four cognitive trajectories. These predefined clusters were used to examine whether hearing measures (4PTA, ACT, HINT) predicted cognitive outcomes and brain volume.

**Findings:**

The combination of PTA and HINT together showed the strongest contribution to classifying previously defined cognitive trajectory groups (p = 0.022). An XGBoost model with SHapley Additive exPlanations (SHAP) values identified bilaterally combined temporal lobe volume as key MRI feature influencing ACT, whereas cerebellum and fourth ventricle were key MRI features influencing HINT.

**Interpretation:**

These findings highlight complex hearing–cognition–brain interactions and support integrating diverse auditory measures into cognitive aging research.

## Introduction

Age-related hearing loss (ARHL) is one of the most common sensory impairments in older adults, and its impact extends far beyond reduced audibility. As the global population ages, an increasing number of individuals experience difficulties understanding speech–especially in noisy, everyday social environments increase as well. Currently ARHL affects more than 65% of adults over 60 years of age (World Health Organizati on, 2021) and is associated with communication difficulties leading to reduced social participation, strained relationships, loneliness, and eventual decline in mental wellbeing (Lin, 2011; Slade et al., 2020; Dawes et al., 2015; Maharani et al., 2019). Moreover, hearing loss has been identified as the largest potentially modifiable risk factor for dementia (Livingston et al., 2017).

Large cohort studies consistently show that the risk of cognitive decline and dementia increases with the severity of hearing loss (Lin, 2011; Lin et al., 2014; Deal et al., 2017a; Gurgel et al., 2014; Windle et al., 2023). Most of this evidence relies on pure-tone audiometry (PTA), which remains the clinical gold standard. However, PTA does not capture speech intelligibility or hearing-in-noise—abilities that are essential for successful daily communication. Measures such as the Audible Contrast Threshold (ACT) (Zaar et al., 2024) and the Hearing-in-Noise Test (HINT) (Nilsson et al., 1994; Soli and Wong, 2008) therefore offer important complementary insight into real-world hearing difficulties. The ACT test represents a novel and clinically relevant approach for investigating how age- or disease-related hearing loss may contribute to cognitive deterioration. Unlike conventional audiometric measures, the ACT test employs temporally modulated noise stimuli to quantify the minimum level of auditory contrast required for an individual to reliably discriminate between sounds. By providing an objective measure of auditory contrast perception, the ACT test enables more precise tailoring of signal-processing strategies to mitigate these distortions, thereby addressing limitations of amplification-only approaches and potentially reducing the downstream cognitive load associated with degraded auditory input. In addition to the ACT, speech-in-noise performance can be further characterized using HINT, which provides a complementary, functional assessment of real-world listening ability and of real-world speech understanding. When combined with ACT results, HINT offers a concise and functionally relevant assessment of speech-in-noise performance. Reduced auditory input in ARHL increases the cognitive effort required for listening, diverting resources away from other cognitive processes (Slade et al., 2020). While hearing aids can mitigate this burden and are associated with better cognitive outcomes (Lin, 2011; Deal et al., 2017b; Rönnberg et al., 2014), many users report limited benefit in noisy environments (Lopez-Poveda et al., 2017; Nuesse et al., 2018), which may contribute to continued social withdrawal.

Neuroimaging studies suggest that ARHL may be linked to regional brain atrophy (Jafari et al., 2021), although findings remain inconsistent. Previous longitudinal studies report accelerated volume loss in individuals with hearing impairment (Lin et al., 2014), whereas others find no direct correspondence between hearing loss and brain morphology change (Eckert et al., 2019). Thus, the neural underpinnings of ARHL, particularly those related to speech-in-noise processing, remain unclear.

Although ARHL is well established as a risk factor for cognitive decline, it remains unclear how deficits in speech intelligibility and hearing-in-noise contribute to early cognitive changes and brain morphological changes. Importantly, newer clinically relevant measures of hearing ability in noise, such as the ACT and the HINT, have not yet been evaluated in studies of cognitive decline and brain volume, representing a key knowledge gap. The novelty of the present study resides in its systematic integration of advanced auditory assessments with comprehensive cognitive measures and neuroimaging outcomes. Accordingly, this study aims to determine whether hearing-in-noise performance, assessed using ACT and HINT alongside PTA, predict cognitive decline. Furthermore, we examine associations between hearing ability and regional brain volumes closely related to auditory processing in participants from a Danish Male Birth Cohort.

## Materials

### Study design and participants

**FIGURE 1:**
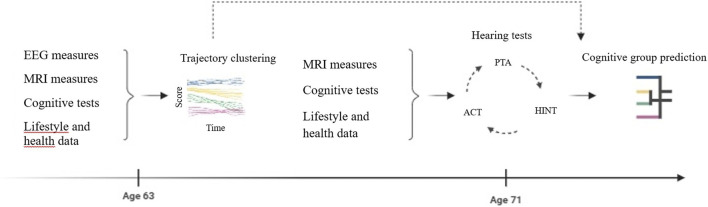
Study design. Participants were randomly recruited from the Metropolit 1953 Danish Male Birth Cohort and underwent baseline testing at age 63. Using deep learning and artificial intelligence, the cohort was clustered into four distinct cognitive trajectories based on longitudinal cognitive performance, MRI, EEG, and lifestyle and health data (Mehdipour Ghazi et al., 2025). At age 71 participants carried out similar test batteries at age 63 including hearing tests to investigate how hearing relates to cognitive outcomes and regional brain volume within these clusters. *EEG*, electroencephalography. *MRI*, Magnetic Resonance Imaging. *ACT*, Audible Contrast Threshold. *PTA*, pure tone audiometry. *HINT*, hearing-in-noise test.

The participants were randomly recruited from the prospective Metropolit 1953 Danish Male Birth Cohort, established in 1965 and comprising all boys born in 1953 in the Copenhagen metropolitan area, whose members have been longitudinally followed throughout life with repeated assessments of cognitive health and multimodal phenotyping (Osler et al., 2004). All participants had a prior visit at the age of 63 ± 1 year (recruited in 2015–2018), including the same cognitive test battery as applied in this study. In our previous work (Mehdipour Ghazi et al., 2025), we applied machine learning (ML) algorithms to identify the influence of individual, familial, and socioeconomic risk factors for cognitive decline within the cohort. In our previous study, we clustered the cohort into four distinct cognitive trajectories based on risk factors, cognitive performance, MRI, and EEG data. These preidentified clusters (1: low-stable and low-declining; 2: high-stable and high-declining) were then consolidated into two by merging the stable and declining categories. These two groups were then used to examine how different aspects of hearing tests, PTA, ACT, HINT, could predict these predefined clustered groups, associated with cognitive aging profiles. Study design is presented in Figure 1. For the current prospective cohort study, a total of 51 elderly men (70.5 ± 0.5 years) were included from the Metropolit 1953 Danish Male Birth Cohort. Exclusion criteria were known medical conditions such as neurodegenerative or major psychiatric disorders, dementia, major brain lesions, alcohol or drug abuse. All participants gave written consent after receiving both verbal and written information about the study. The project was approved by the local ethical committee (Region Zealand, Denmark, SJ-1036) and conducted according to the Declaration of Helsinki.

### Procedures

#### Cognitive tests

To evaluate cognitive functioning, a comprehensive neuropsychological battery was administered, incorporating tasks from the Cambridge Neuropsychological Test Automated Battery (CANTAB® [Cognitive assessment software], Cambridge Cognition (2018), all rights reserved. www.cantab.com) alongside established standardized cognitive screening instruments.

Within the CANTAB framework, motor screening (MOT) was used to ensure adequate sensorimotor function and to establish a baseline level of task performance for subsequent measures. Spatial recognition memory (SRM) assessed the ability to encode and retrieve spatial information, whereas pattern recognition memory (PRM) examined visual recognition memory for previously encountered stimuli. Paired-associate learning (PAL) was employed to evaluate episodic memory formation and associative learning capacity. The Stockings of Cambridge (SOC) task measured higher-order executive functions, particularly planning efficiency and problem-solving ability. Reaction time (RTI) indexed psychomotor speed and information processing efficiency, while the rapid visual information processing (RVP) task assessed sustained attention and aspects of working memory under continuous performance demands.

In addition to the CANTAB battery, several complementary neuropsychological instruments were included. The Addenbrooke’s Cognitive Examination (ACE) provided a broad multidomain assessment of global cognitive status, while the Mini-Mental State Examination (MMSE) served as a brief screening measure for general cognitive impairment. The Symbol Digit Modalities Test (SDMT) evaluated attention, visual scanning efficiency, and psychomotor speed. Executive control and cognitive flexibility were assessed using the Trail Making Tests (TMT) Parts A and B, which respectively index processing speed and set-shifting ability. Finally, verbal learning and memory performance were examined using a 15-word paired-associate recall paradigm, capturing both immediate retention and delayed retrieval capacity.

#### Hearing assessments

Three different tests were used for the hearing assessment. Hearing sensitivity was measured with PTA calculating a speech-frequency pure tone average of air-conduction thresholds at 0.25, 0.5, 1, 1.5, 2, 3, 4, 6 and 8 kHz. Hearing impairment was defined as a 4PTA (0.5, 1, 2 and 4 kHz) > 20 dB in the better ear. ACT was used to evaluate sensitivity to changes in sound features. The participants were presented bilaterally with a continuous audibility-corrected noise via headphones and instructed to press a pushbutton whenever they heard a target sound in the noise. Hearing threshold was established using a three-out-of-five criterion tracking procedure. Based on normative data, a normalized Contrast Level (in dB nCL) scale was defined, where 0 ± 4 dB nCL corresponded to normal hearing thresholds whereas an elevated dB nCL level signified the degree of audible contrast loss (Zaar et al., 2024). For measuring speech perception in noisy environments HINT was carried out using a single front-placed loudspeaker for both the speech and noise sources (Nilsson et al., 1994; Soli and Wong, 2008; Nielsen and Dau, 2011). Before the test lists, participants were provided with a practice run on a practice list (randomly chosen from a different set) to acclimatize them to the test settings.

The signal-to-noise ratio where 50% of the presented sentences were correctly repeated by the participants were used as the speech recognition threshold in noise (SRT_N_). Participants using hearing aids performed the familiarization list and the two test list without hearing aids and thereafter performed another list with the use of hearing aids. HINT results for hearing aid users are presented with the use of hearing aids.

#### Neuroimaging

Structural brain data were collected to evaluate changes in regional brain volume using high-resolution magnetic resonance imaging (MRI). The MRI scans were performed on a 3.0-T MRI scanner (MAGNETOM Vida; Siemens Healthcare) with a 64-channel phased array head coil since 2023 and the previous scans between 2008 and 2023 on a 3T Philips Achieva (240 × 256 × 180, 0.7 mm isotropic). MRI volumetry was performed using FAST-AID Brain (Mehdipour Ghazi and Nielsen, 2025) on T1-weighted scans. Regional volumes from left and a right hemisphere were merged and corrected for intracranial volume (ICV) -https://link.springer.com/chapter/10.1007/978-3-031-95911-0_12 -, yielding 68 brain regions including hippocampus, ventricles, white matter, and CSF. The 68 regions were obtained by combining left and right compartment volumes into single bilateral regions. This step was performed to reduce dimensionality and improve the stability of estimates given the limited sample size relative to the number of features, thereby reducing the risk of overfitting and improving robustness of subsequent analyses.

#### Lifestyle and health data

Participants completed comprehensive questionnaires regarding lifestyle, mood and health as well as questionnaires detailing their hearing status, use of hearing aids, and duration of hearing aid usage. These data were analyzed to account for potential confounding factors such as physical activity, smoking, alcohol consumption, and co-morbidities.

### Statistical analysis

To investigate the relationship between hearing function and cognitive performance, paired samples *t*-tests were performed to assess changes in cognitive outcomes over the 8-year follow-up period. Participants were subsequently categorized into three hearing-status groups based on their hearing assessments: normal hearing (NH; 4PTA <20 dB HL), hearing impairment (HI; 4PTA ≥20 dB HL), and hearing-aid users (HA; bilateral hearing-aid use in daily life). Group differences in cognitive performance were initially examined using one-way analysis of variance (ANOVA), with hearing status as the between-subjects factor. Descriptive data are reported as mean ± standard deviation (SD). Additionally, multiple regression models were used to evaluate the predictive value and statistical significance of various clinical and cognitive variables. Brain imaging data were analyzed to assess correlations between regional brain volumes and both hearing impairment and cognitive decline. Additionally, longitudinal data were utilized to quantify changes in cognitive function and the extent of neurodegeneration over time, allowing for a more comprehensive understanding of progressive decline in our study cohort.

#### Model development

To address the complexity of cognitive decline, we employed a temporal k-means clustering algorithm combined with multivariate dynamic time warping (DTW) distance. This allowed us to group individuals based on the overall shape of their cognitive trajectories, even if the sequences differed in length or had missing values. Cognitive performance across several domains: Reaction time, processing speed, attention, executive function (CANTAB), IQ (IST), visuospatial abilities (ACE, CANTAB) and language abilities (ACE) was first summarized, followed by computation of DTW distances between participants (Mehdipour Ghazi et al., 2025). The resulting similarity matrix enabled clustering into homogeneous groups, with the optimal number of clusters (four; 1: low-stable and 3: low-declining, 2: high-stable, 4: high-declining) selected by minimizing the Davies-Bouldin index, which quantifies cluster compactness and separation. To evaluate the predictive potential of hearing, we conducted logistic regression analyses with interaction terms, using four-frequency PTA (4PTA), HINT, and ACT as predictors. The binary outcome variable was cognitive performance group (low vs. high). This approach enables preliminary evaluation of the predictive strength of auditory assessments in distinguishing cognitive status among older adults. Furthermore, we developed an eXtreme Gradient Boosting (XGBoost) algorithm to model ACT and HINT scores. XGBoost is efficient in handling high-dimensional datasets and achieving high predictive accuracy through an ensemble of decision trees. It uses gradient boosting to optimize predictions efficiently, making it very effective for both regression and classification problems on structured datasets (Chen and Guestrin, 2016). Predictor variables included: T1-MRI measures from 49 subjects with 68 regional volumes normalized with ICV.

#### SHAP value visualization

To interpret the trained model, we utilized SHapley Additive exPlanations (SHAP) values (Lundberg and Lee, 2017). SHAP values provide insights into how each input variable contributes to the model’s predictions. This method allows a comprehensive understanding of feature importance and the impact of each predictor on the output.

SHAP values are quantified as the log-odds (logit), i.e., log (p/(1 - p)), where p is the probability of an event happening. The SHAP values are visualized in SHAP summary plots to illustrate the contribution of each input variable to the model’s output. This visualization aids in identifying influential features and understanding their effects on the predicted outcomes. In the summary plots, each point represents an observation, positioned horizontally according to its SHAP value (impact on the model output) and colored by the feature value (blue = low, red = high). Features with wider horizontal spreads have greater influence, while the color gradient indicates whether higher or lower feature values increase or decrease the predicted outcome. This allows a compact view of feature importance, effect direction, and consistency across the dataset.

## Results

### Participant characteristics

Participants were recruited in 2024 from the Metropolit 1953 Danish Male Birth Cohort. In total 51 participants were included in the analysis out of which only 49 had MRIs, 48 had ACT plus HINT and only 46 had been clustered into groups. A total of fifteen participants were classified and numbered in the present study as low performers (cluster 1: low-stable, 3: low-declining), whereas 31 were categorized as high performers (cluster 2: high-stable, 4: high-declining) (Mehdipour Ghazi et al., 2025).

### Cognitive function

**FIGURE 2:**
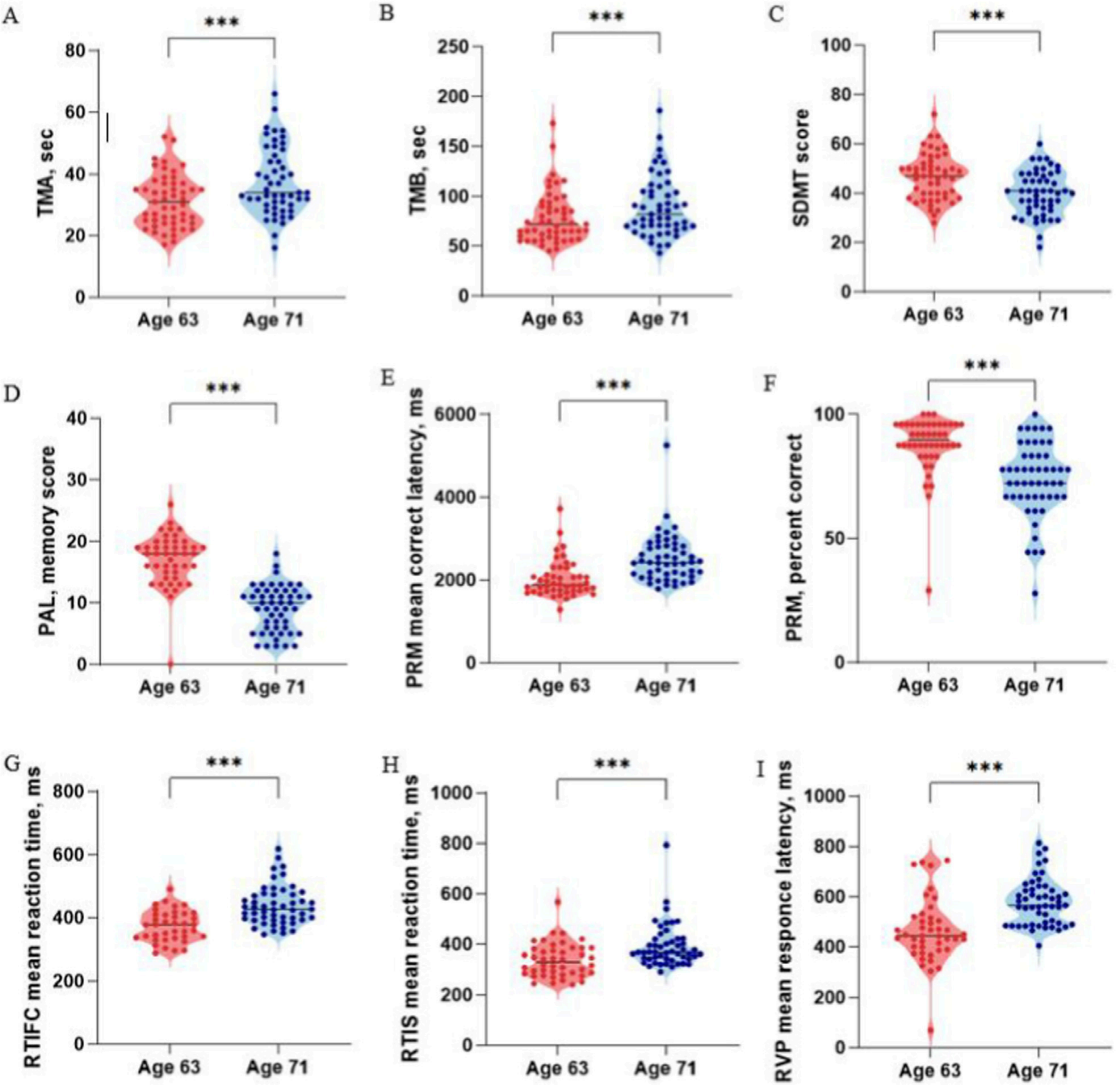
Nine panel violin plot figure compares cognitive performance between the same participants age sixty-three and seventy-one. Panels display scores or times for processing speed; TMA/ TMB (trail making test) SDMT (Symbol Digit Modalities Test) **(A–C)**, memory; PAL (Paired Associates Learning) **(D)**, PRM latency (pattern recognition memory) **(E)**, PRM percent correct **(F)**, RTIFC (Reaction time) and RTIS mean reaction times **(G, H)**, and RVP (Rapid Visual Information Processing) mean response latency **(I)**. Data points and distributions show significant age-related decline (indicated by asterisks). As participants grew older, they tended to show slower reaction times, poorer memory scores, and decreased accuracy.

From age 63 to 71, notable declines were detected in several cognitive domains, particularly in processing speed (TMA +6.2 s (p < 0.001), TMB +11.4 s (p < 0.001), and SDMT –9 score (p < 0.001)), reaction time (simple +54.7 ms (p < 0.001) and five-choice +62.8 ms (p < 0.001)), short-term memory as measured by PAL first attempt memory score −8 score (p < 0.001)), and pattern recognition memory (PRM mean correct latency +497.1 ms (p < 0.001) and 18.75% less correct answers (p < 0.001)), and visual processing (RVP mean response latency +116.3 ms (p < 0.001)) (Figure 2). No age-related changes from age 63 to 72 were obtained in MMSE, ACE, VPA or IST-200R. In addition, no significant difference in cognitive function was observed between hearing groups (Table 1).

**TABLE 1:** *MMSE*: mini mental state examination, *ACE:* Addenbrooke’s Cognitive Assessment, *TMA:* trail making A, *TMB:* trail making B, *SDMT:* Symbol-Digit Modalities Test, *VPA:* Verbal Paired Associates, *IST-2000R:* Intelligence Structur Test.

Cogtitive tests	Age 63 (n = 51)	Age 71 (n = 51)	p-value	NH at age 71 (n = 19)	IH at age 71 (n = 21)	HA at age 71 (n = 11)	p-value
MMSE, score	29.2 ± 1.2	29.2 ± 1.1	0.841	29.4 ± 0.8	28.9 ± 1.3	29.5 ± 1.2	0.206
ACE, score	92.7 ± 5.6	92.3 ± 6.4	0.563	94.2 ± 4.2	90.5 ± 7.1	91.9 ± 7.8	0.196
TMA, sec	31.3 ± 8.5	37.5 ± 11.0	<0.001***	36.9 ± 11.5	37.0 ± 11.1	39.3 ± 10.6	0.836
TMB, sec	78.4 ± 26.0	89.8 ± 30.4	<0.001***	90.5 ± 38.4	90.7 ± 25.1	86.7 ± 26.5	0.935
SDMT, score	49.0 ± 9.3	40.1 ± 8.9	<0.001***	43.4 ± 8.8	38.3 ± 9.6	37.6 ± 6.0	0.113
VPA, error	15.0 ± 10.2	16.2 ± 11.6	0.297	16.4 ± 10.8	17.0 ± 11.2	14.3 + 14.3	0.793
VPA 1h retention, error	6.1 ± 4.0	6.3 ± 4.1	0.748	6.4 ± 3.5	6.4 ± 4.5	5.8 ± 4.4	0.893
IST-2000R, score	29.8 ± 11.5	28.0 ± 11.3	0.334	29.9 ± 10.6	27.0 ± 12.2	31.0 ± 11.0	0.589

***p < 0.001. NH: normal hearing, IH: impaired hearing, HA: hearing aid users.

#### Hearing ability

**FIGURE 3:**
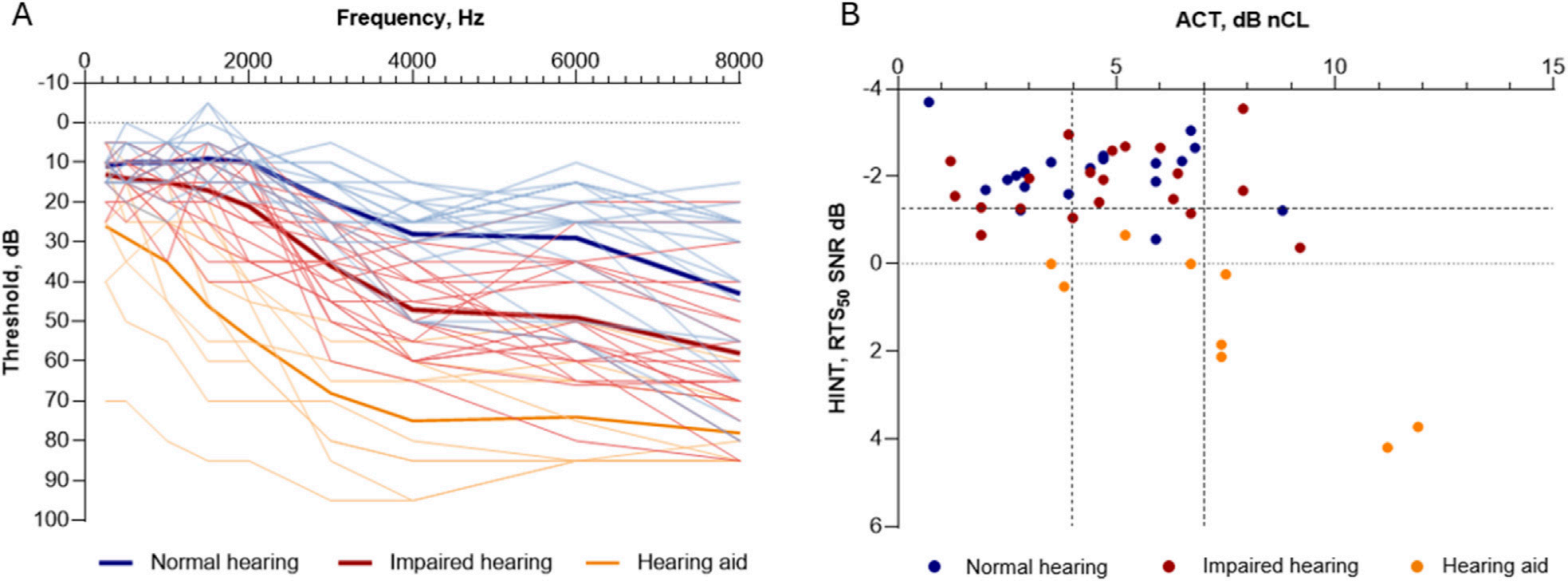
**(A)** Pure-tone Audiometry (PTA) individual and group mean audiograms for normal hearing individuals (blue lines), hearing impairment (red lines) and hearing aid users (orange lines) **(B)** The diversity of hearing profiles across Audible Contrast Threshold (ACT), Hearing-in-Noise Test (HINT), and 4PTA. HINT score is expressed as reception speech threshold 50% (RTS_50_) corresponding to the signal-to-noise ratio (SNR) in dB at which the participant correctly understood 50% of the speech. ACT is expressed as dB normalized Contrast Level (nCL). 4PTA hearing threshold is indicated by color whereas blue indicates normal hearing, red indicates impaired hearing, and orange indicates hearing aid users. Vertical dashed lines illustrate mild and moderate contrast loss, respectively and horizontal dashed line illustrate population mean of HINT.

4PTA thresholds were calculated across 0.5, 1, 2, and 4 kHz. Based on 4PTA 19 participants (37.3%) had normal hearing defined as 4PTA <20 dB, with an average threshold of 15 ± 3 dB and 21 participants (41.1%) had hearing impairment defined as 4PTA >20 dB, with an average threshold 25.8 ± 3.7 dB. A total of 11 participants (21.6%) were regular hearing aid users, using bilateral hearing aid for daily life (n = 10) or cochlear implant (n = 1) (> 3 years and ≥8 h/day). The 4PTA average threshold for the hearing aid users was 49.3 ± 14.8 dB. Individual and group mean audiograms are presented in Figure 3A. The relation between 4PTA, ACT and HINT is illustrated in Figure 3B. The mean SRT_N_ for the full cohort on the HINT test was − 1.34 ± 1.63 dB SNR, therefore normal HINT threshold was defined as under − 1.34 dB SNR. Normal hearing in ACT was defined as hearing threshold under 4 dB. A total of eight participants exhibited normal hearing across all three auditory assessments. Whereas five participants demonstrated impaired hearing on all three assessments; notably, 80% of this latter group were hearing aid users. Among the 19 participants with normal 4PTA thresholds, 10 participants (53%) demonstrated contrast loss on the ACT, and three of them also showed impaired performance on the HINT. Conversely, 13 participants (62%) with impaired 4PTA thresholds performed within the normal range on the HINT, and eight participants with impaired 4PTA also showed normal performance on the ACT. Additionally, two hearing aid users demonstrated normal ACT threshold.

#### Hearing ability and interaction effects in predicting cognitive decline

**FIGURE 4:**
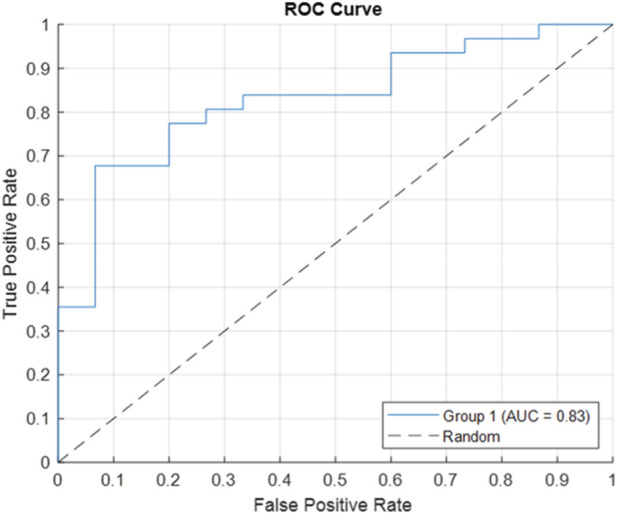
Receiver Operating Characteristic (ROC) curve for interaction term x1:x3 (4PTA:HINT), showing the relationship between the true positive rate and false positive rate across different classification thresholds. The solid line represents the model performance with an area under the curve (AUC) of 0.83, while the dashed diagonal line represents random classification. AUC reflects model performance within the analyzed sample and should not be interpreted as an externally validated estimate of predictive accuracy.

A logistic regression model with interaction terms was used to predict cognitive decline, with predictors including all three hearing tests, as well as their interactions. The model included the main effects [x1 (4PTA), x2 (ACT), x3 (HINT)] and interactions [x1:x2, x1:x3, x2:x3]. The interaction between 4PTA and HINT was statistically significant (p = 0.022), while none of the other main effects or interactions reached significance (all p > 0.05). Figure 4 shows important features identified, as assessed by the absolute values of regression coefficient; the interaction term x1:x3 (4PTA:HINT) had the highest influence (4.1262), followed by HINT alone (2.6157) and 4PTA alone (0.7354) (Figure 4). Additionally, we report bootstrap confidence intervals for the hearing-model coefficients and assess whether the 4PTA × HINT interaction remains among the dominant terms across 1,000 bootstrap resamples (Table 2). In addition, the bootstrap ROC-AUC of 0.870 ± 0.061 remains comparable with the full-sample ROC-AUC of 0.830. For the XGBoost models, we added a SHAP stability analysis in which feature importance rankings were recomputed across 1,000 bootstrap resamples and summarized by median values. We assessed whether the same anatomical regions repeatedly remain important (Table 3).

**TABLE 2:** Logistic regression model coefficients.

Feature	Full sample	Bootstrap (median)	95% bootstrap CI	Bootstrap *p* < 0.05 frequency
4PTA	−0.735	−0.731	[-3.465, 1.919]	134
ACT	0.255	0.457	[-0.713, 2.715]	64
HINT	−2.616	−3.555	[-13.216, −0.621]	328
4PTA × ACT	−0.516	−0.585	[-4.352, 2.417]	48
4PTA × HINT	4.126 (*p* < 0.05)	5.129	[1.683, 20.329]	637
ACT × HINT	−1.123	−1.192	[-6.854, 3.583]	83

**TABLE 3:** SHAP features importance ranking calculated based on mean absolute SHAP values.

Feature	Full sample HINT	Bootstrap (median) HINT	Full sample ACT-Impaired	Bootstrap (median) ACT-impaired
CSF	14	6	3	4
Basal ganglia	5	7	5	6
Temporal lobe	8	7	1	2
Cingulate	7	6.5	12	10
Insula	6	8	14	11
Frontal lobe	3	4	13	9
Parietal lobe	4	10	11	8
Occipital lobe	13	11	8	8
Cerebral WM	11	9	10	9
Cerebellum	2	6	2	3
Cranial cavity	10	8	7	10
CSF medial	9	8	4	6
Forth ventricle	1	2	6	8
Brain stem	12	12	9	9

#### Key MRI predictors of hearing-in-noise identified by SHAP analysis

**FIGURE 5:**
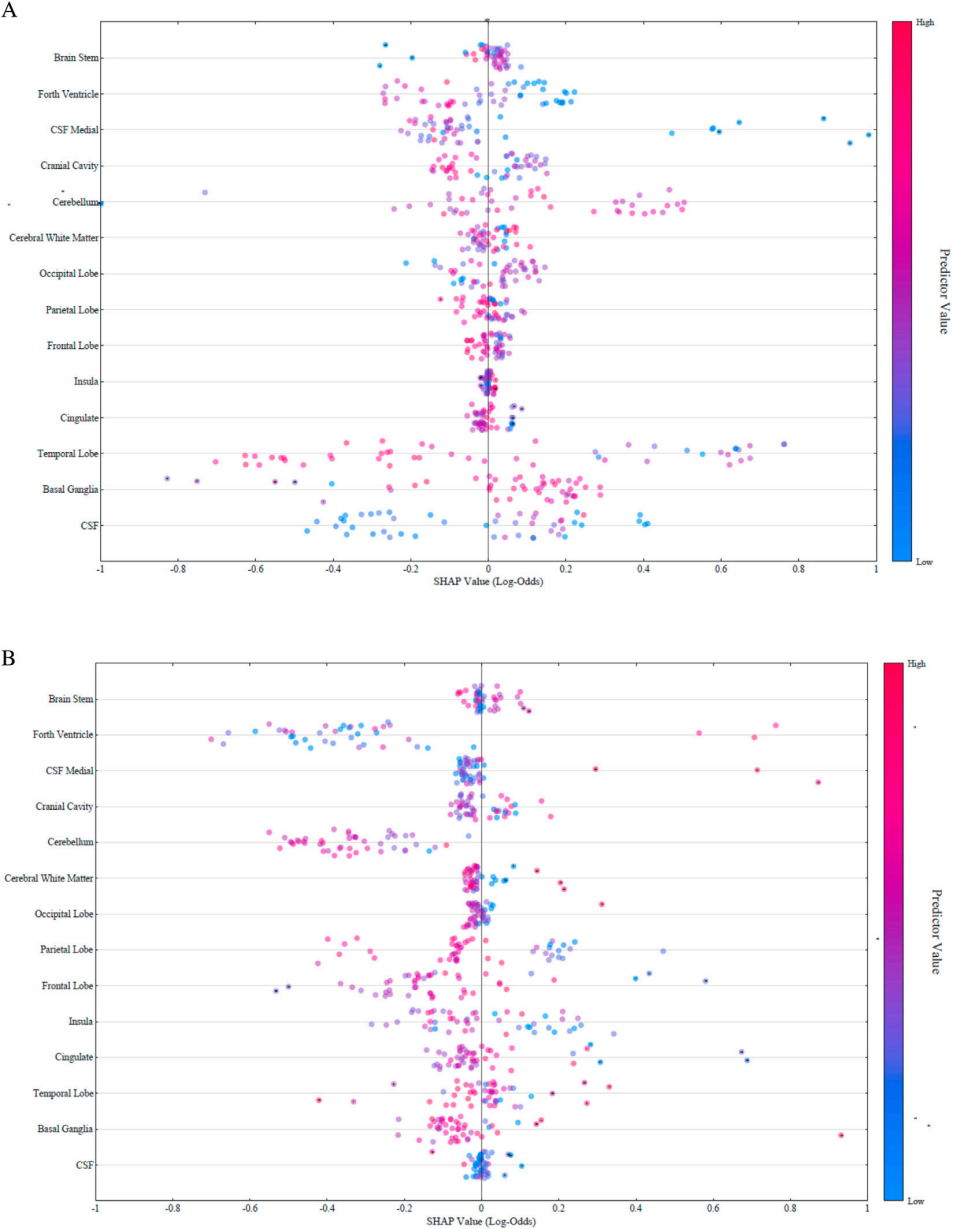
SHapley Additive exPlanations (SHAP) value summary plots showing the contribution of brain region predictors to the XGBoost (eXtreme Gradient Boosting) model classification of impaired Audible Contrast Threshold (ACT >4.0 dB) **(A)** and HINT performance **(B)**. Each dot represents one data point, and the color shows whether the feature value is high (red) or low (blue). Dots to the right mean the feature increases the prediction of the XGBoost model, while dots to the left mean they decrease it. The farther a dot is from the center, the more influence that feature has on the output. *CSF: cerebrospinal fluid.*

The trained XGBoost models for ACT classification and HINT regression were used for feature importance analysis. Interpretation of SHAP values revealed key insights into feature contributions in terms of neuroimaging biomarkers as shown in Figure 5. (Figure 5). The SHAP analysis indicates that volumetric measures across multiple brain regions contribute to the prediction of the model of hearing-in-noise outcome. Overall, both positive and negative SHAP values are distributed across nearly all regions, suggesting a complex and non-linear association between brain morphology and the auditory function captured by the ACT and HINT measure. However, SHAP analysis identified temporal lobe volume and cerebrospinal fluid (CSF) measures as the primary contributors for impaired ACT score (Figure 5A). Lower temporal lobe volume and higher CSF volume were associated with increased probability of impaired ACT score. Secondary contributions were observed for the basal ganglia and cerebellum, which showed more moderate and heterogeneous effects on the output of the model. Additionally, SHAP analyses for HINT indicated higher volumes of the cerebellum, frontal lobe, and parietal lobe were associated with lower HINT scores. Furthermore, low volume of the fourth ventricle was linked to lower HINT score. Other cortical regions exhibited limited influence, with SHAP values centered near zero (Figure 5B).

## Discussion

The major findings of the present study were that 4PTA and HINT performance constitute the most reliable indicators of cognitive decline in older adults. Importantly, the interaction between 4PTA and HINT was identified as a significant contributor, emphasizing the critical role of evaluating both peripheral auditory sensitivity and central auditory processing in the assessment of cognitive impairment risk. Bootstrap analysis of the hearing-based logistic regression model showed that the 4PTA × HINT interaction remained among the largest model terms across resamples, supporting the full-sample finding that combined peripheral hearing sensitivity and speech-in-noise performance contributed most strongly to cognitive group separation. Recent studies increasingly support a multidimensional approach to characterizing age-related hearing and its relationship with cognitive decline. While pure-tone average (PTA/4PTA) remains the most widely used clinical measure of peripheral hearing sensitivity, speech-in-noise tests such as the Hearing-in-Noise Test (HINT) are frequently added to better capture real-world auditory function (Wang et al., 2026; Jiang et al., 2022). More recently, research has begun to integrate additional psychoacoustic measures, including the Audible Contrast Threshold (ACT), to reflect higher-level auditory processing beyond audibility and speech recognition. Although combinations of PTA and speech-in-noise measures are now relatively common in cognitive ageing research, the simultaneous use of 4PTA, HINT, and ACT remains uncommon and is mainly seen in emerging multimodal or predictive modelling studies (Sanchez-Lopez et al., 2026). This integrated approach may provide a more comprehensive auditory profile, potentially improving the prediction of cognitive decline compared with single-measure assessments, but further validation in larger and more diverse cohorts is still needed. Another important finding was that our SHAP stability analysis confirmed the robustness of the main anatomical contributors identified in the full-sample XGBoost models with the same key contributors recurring across bootstrap resamples. For ACT, temporal lobe, cerebellum, and CSF-related measures were repeatedly ranked among the most influential features. For HINT, frontal lobe, cerebellar, and fourth-ventricle measures were repeatedly among the top-ranked contributors. Exact rankings varied across resamples, indicating that the findings should be interpreted as exploratory and hypothesis-generating rather than as definitive biomarkers.

Together, these findings suggest that hearing in noisy environments reflects multiple interacting neural and sensory factors, which may partly explain the considerable interindividual variability observed in both ACT and HINT outcomes. Our findings are in line with earlier studies (Wu et al., 2022), suggesting that cognitive factors can help explain observed differences and improve aided outcome prediction. Our findings are also consistent with prior research indicating that comprehension profiles reflect underlying cognitive mechanisms and processes (Ellis et al., 2025; Cordonier et al., 2023). Furthermore, studies investigating cognitive ability and hearing outcomes in middle-aged and older adults have identified cognition as an important factor contributing to individual differences in auditory performance and rehabilitation outcomes (Helfer et al., 2024), cognitive ability and hearing outcomes in middle-aged and older adults, similarly emphasized the role of cognition as a key contributor to variability in auditory performance and rehabilitation outcomes. The present study further illustrates the potential utility of SHAP-based explainable machine learning for exploring complex relationships between hearing outcomes and brain structure. Given the large number of MRI-derived features included in the analyses, SHAP values facilitated the interpretation of model outputs by quantifying the relative contribution of individual variables to hearing predictions. This approach provided greater transparency regarding how the model incorporated information across multiple domains. While the findings should be interpreted within the context of the study design and sample size, the use of explainable machine learning offers a useful framework for investigating the relative importance of neural factors associated with hearing in aging populations. More broadly, the integration of multimodal biomarkers with interpretable machine-learning approaches may help inform future studies seeking better understanding the relationships among hearing, cognition, and brain aging. Therefore, the results should be interpreted as characterizing associative patterns within this cohort rather than as population-level estimates. In this context, machine learning methods such as XGBoost were used to explore multivariate relationships across modalities that may not be fully captured by univariate statistical models. These analyses are intended to complement traditional inference by highlighting consistent feature contributions within the dataset, rather than to provide generalizable predictive performance.

Even though our results indicate a complex and non-linear relationship between brain morphology and auditory performance as assessed by the ACT and HINT measures, several region-specific associations emerged. Specifically, reduced temporal lobe volume and increased CSF volume were associated with a higher likelihood of impaired ACT scores. In contrast, greater cerebellar volume, together with lower fourth ventricle volume, both indicative of preserved deep brain structures, was associated with better HINT performance, reflected by lower (i.e., better) HINT scores.

However, no statistically significant differences were observed between hearing aid users, non-users, and individuals with normal hearing across the primary hearing group comparisons. In contrast, longitudinal cognitive analyses revealed a significant decline between ages 63 and 71 in processing speed, reaction time, and short-term memory performance, indicating measurable age-related changes in specific cognitive domains over the study period. These findings are consistent with prior work showing that age-related hearing impairment is associated with structural alterations in temporal cortical regions and broader brain atrophy patterns (Kirschen and Leaver, 2024; Khandalavala et al., 2024; Svob et al., 2024).

These brain areas participate in networks supporting auditory processing, multimodal integration, and cognitive control, suggesting that subtle volumetric changes may reflect underlying neural vulnerability influencing both auditory and cognitive aging trajectories (Mihai et al., 2021; Lima et al., 2016). Involvement of temporal lobe and cerebellum as well as frontal and parietal lobe volume support the concept of global temporal processing, sensorimotor integration, working memory, and predictive coding mechanisms that are increasingly recognized as fundamental to speech perception in complex acoustic environments. In contrast, enlargement or extension of the fourth ventricle–a likely marker of regional atrophy–proved highly predictive of poor sentence understanding in noise. This relationship reinforces the view that neurodegenerative changes in brainstem-cerebellar systems play a meaningful role in the auditory difficulties experienced by older adults. The novelty of this study lies in its use of the ACT as a complementary measure to traditional assessments of hearing ability. Unlike conventional audiometry tests, the ACT specifically evaluates auditory contrast perception, allowing for a more targeted investigation of auditory processing deficits. Importantly, our analysis using SHAP values revealed that the regional brain atrophy patterns contributing to impaired ACT differed from those predicting performance on the HINT. While HINT performance, reflecting sentence comprehension in noisy environments, was associated with the cerebellar volume, ACT performance was more closely linked to structural volume variations within the temporal lobe, highlighting the critical role of these regions in fine-grained auditory discrimination. These findings suggest that ACT captures aspects of auditory-cognitive processing that are not fully represented by general cortical atrophy, offering a more region-specific insight into the neural correlates of auditory perception. This distinction underscores the potential of ACT as a sensitive tool for detecting early auditory-related deficits in populations at risk for brain aging (Danielsson et al., 2019).

4PTA and HINT together provide stronger predictive value for cognitive decline than either measure alone. This is likely because they capture complementary aspects of auditory functioning, with 4PTA reflecting peripheral hearing sensitivity and HINT indexing central auditory speech-in-noise processing. Their combined use therefore improves predictive accuracy by incorporating greater heterogeneity in auditory processing mechanisms relevant to cognitive decline, compared with reliance on a single measure. These findings align with accumulating evidence linking ARHL to decreased cognitive outcomes and suggest that combined auditory measures may serve as sensitive indicators for distinguishing between cognitively high- and low-performing individuals (Mihai et al., 2021; Lima et al., 2016). Consistent with prior work, early declines in executive function and processing speed, domain indicators that often change first in cognitively normal but aging populations, appear particularly influential in shaping auditory task performance, especially under challenging conditions (O’Brien et al., 2020; Danielsson et al., 2019; Ferguson et al., 2021). Finally, the use of hearing aids does not necessarily correspond to improved functional hearing in everyday listening situations, particularly in noisy or acoustically complex environments. In the present study, no conclusions can be drawn regarding the effect of hearing aid use in these settings. Future research incorporating clinically relevant measures of hearing aid use is needed to better understand the benefits from hearing aids.

## Conclusion

In conclusion, the results suggest that different hearing measures may capture distinct aspects of age-related auditory decline and brain aging. Specifically, impaired performance on HINT and ACT may reflect neural and cognitive processes beyond peripheral hearing sensitivity alone, whereas elevated PTA thresholds in the absence of impaired HINT or ACT performance may be more indicative of predominantly peripheral auditory decline. Although these interpretations require confirmation in larger longitudinal cohorts, they offer a potential framework for understanding why individuals with apparently similar degrees of hearing loss may follow different cognitive and neurobiological trajectories.

## Limitation

A limitation of the present study is the relatively small sample size, which restricts the strength of conclusions regarding predictive performance and the generalizability of the findings. Importantly, the primary aim of this work was not to develop or validate a highly accurate predictive model, but rather to explore the relationships between multimodal factors and cognitive cluster membership and to identify variables that may contribute to classification. Accordingly, the focus was placed on the interpretation of predictor importance and the characterization of potentially relevant factors rather than on prediction accuracy itself. Given the limited sample size, feature importance estimates should be interpreted with caution and considered exploratory. Consequently, the identified predictors should be viewed as hypothesis-generating and as providing insight into potential mechanisms underlying cognitive heterogeneity, rather than as definitive biomarkers or validated predictors. Future studies with larger and more diverse cohorts are needed to confirm these findings and assess their broader applicability.

## Data Availability

The datasets presented in this article are not readily available because Individual participant data cannot be shared as the informed consent signed by patients does not allow data sharing. Requests to access the datasets should be directed to Krisztina Benedek, krben@regionsjaelland.dk.

## References

[B1] Chen T. Guestrin C. (2016). XGBoost: A Scalable Tree Boosting System. New York, NY: Association for Computing Machinery, 785–794. 10.1145/2939672.2939785

[B2] Cordonier N. Fossard M. Tillé Y. Champagne-Lavau M. (2023). Exploring cognitive-pragmatic heterogeneity following acquired brain injury: a cluster analysis of hint comprehension. Am. J. Speech-Language Pathology 32 (6), 2752–2767. 10.1044/2023_AJSLP-22-00389 37707362

[B3] Danielsson H. Humes L. E. Rönnberg J. (2019). Different associations between auditory function and cognition depending on type of auditory function and type of cognition. Ear Hear 40, 1210–1219. 10.1097/AUD.0000000000000700 30807540 PMC6706331

[B4] Dawes P. Emsley R. Cruickshanks K. J. Moore D. R. Fortnum H. Edmondson-Jones M. (2015). Hearing loss and cognition: the role of hearing aids, social isolation and depression. PLoS One 10, 1–9. 10.1371/journal.pone.0119616 25760329 PMC4356542

[B5] Deal J. A. Betz J. Yaffe K. Harris T. Purchase-Helzner E. Satterfield S. (2017a). Hearing impairment and incident dementia and cognitive decline in older adults: the health ABC study. Journals Gerontol. - ser. A Biol. Sci. Med. Sci. 72, 703–709. 10.1093/gerona/glw069 PMC5964742 27071780

[B6] Deal J. A. Albert M. S. Arnold M. Bangdiwala S. I. Chisolm T. Davis S. (2017b). A randomized feasibility pilot trial of hearing treatment for reducing cognitive decline: results from the aging and cognitive health evaluation in elders pilot study. Alzheimer’s Dement. Transl. Res. Clin. Interv. 3, 410–415. 10.1016/j.trci.2017.06.003 29067347 PMC5651440

[B7] Eckert M. A. Vaden K. I. Dubno J. R. (2019). Age-related hearing loss associations with changes in brain morphology. Trends hear. 23, 1–14. 10.1177/2331216519857267 31213143 PMC6585256

[B8] Ellis G. M. Bieber R. Davidson A. Sherlock L. Spencer M. Brungart D. (2025). Improving the predictive strength of better-ear four-frequency pure-tone average with the addition of the tinnitus and hearing survey-hearing subscale. Ear Hear. 46 (4), 909–921. 10.1097/AUD.0000000000001633 39885626

[B9] Ferguson H. J. Brunsdon V. E. A. Bradford E. E. F. (2021). The developmental trajectories of executive function from adolescence to old age. Sci. Rep. 11, 1–17. 10.1038/s41598-020-80866-1 33446798 PMC7809200

[B10] Gurgel R. K. Ward P. D. Schwartz S. Norton M. C. Foster N. L. Tschanz J. T. (2014). Relationship of hearing loss and dementia. Otol. Neurotol. 35, 775–781. 10.1097/mao.0000000000000313 24662628 PMC4024067

[B11] Helfer K. S. Maldonado L. Matthews L. J. Simpson A. N. Dubno J. R. (2024). Extended high-frequency thresholds: associations with demographic and risk factors, cognitive ability, and hearing outcomes in middle-aged and older adults. Ear Hearing 45 (6), 1427–1443. 10.1097/AUD.0000000000001531 38987892 PMC11493509

[B12] Jafari Z. Kolb B. E. Mohajerani M. H. (2021). Age-related hearing loss and cognitive decline: MRI and cellular evidence. Ann. N. Y. Acad. Sci. 1500, 17–33. 10.1111/nyas.14617 34114212

[B13] Jiang K. Armstrong N. M. Agrawal Y. Gross A. L. Schrack J. A. Lin F. R. (2022). Associations of audiometric hearing and speech-in-noise performance with cognitive decline among older adults: the Baltimore longitudinal study of aging (BLSA). Front. Neurol. 13, 1029851. 10.3389/fneur.2022.1029851 36570462 PMC9784219

[B14] Khandalavala K. R. Marinelli J. P. Lohse C. M. Przybelski S. A. Petersen R. C. Vassilaki M. (2024). Neuroimaging characteristics of hearing loss in the Mayo clinic study of aging. Otolaryngol. Head. Neck Surg. 170 (3), 886–895. 10.1002/ohn.583 38018509 PMC10922536

[B15] Kirschen R. M. Leaver A. M. (2024). Hearing function moderates age-related differences in brain morphometry in the HCP aging cohort. Hum. Brain Mapp. 45 (16), e70074. 10.1002/hbm.70074 39540247 PMC11561423

[B16] Lima C. F. Krishnan S. Scott S. K. (2016). Roles of supplementary motor areas in auditory processing and auditory imagery. Trends Neurosci. 39, 527–542. 10.1016/j.tins.2016.06.003 27381836 PMC5441995

[B17] Lin F. R. (2011). Hearing loss and cognition among older adults in the United States. Journals gerontol. - ser. A biol. Sci. Med. Sci. 66 (A), 1131–1136. 10.1093/gerona/glr115 21768501 PMC3172566

[B18] Lin F. R. Ferrucci L. An Y. Goh J. O. Doshi J. Metter E. J. (2014). Association of hearing impairment with brain volume changes in older adults. Neuroimage 90, 84–92. 10.1016/j.neuroimage.2013.12.059 24412398 PMC3951583

[B20] Livingston G. Sommerlad A. Orgeta V. Costafreda S. G. Huntley J. Ames D. (2017). Dementia prevention, intervention, and care. Lancet 390, 2673–2734. 10.1016/S0140-6736(17)31363-6 28735855

[B21] Lopez-Poveda E. A. Johannesen P. T. Pérez-González P. Blanco J. L. Kalluri S. Edwards B. (2017). Predictors of hearing-aid outcomes. Trends hear. 21, 1–28. 10.1177/2331216517730526 28929903 PMC5613846

[B22] Lundberg S. Lee S. I. (2017). A Unified Approach to Interpreting Model Predictions. 4766–4775. 10.48550/arXiv.1705.07874

[B23] Maharani A. Pendleton N. Leroi I. (2019). Hearing impairment, loneliness, social isolation, and cognitive function: longitudinal analysis using English longitudinal study on ageing. Am. J. Geriatr. Psychiatry 27, 1348–1356. 10.1016/j.jagp.2019.07.010 31402088

[B24] Mehdipour Ghazi M. Nielsen M. (2025). FAST-AID brain: fast and accurate segmentation tool using artificial intelligence developed for brain. In: Petersen, J., Dahl, V. A. (eds) Image Analysis. SCIA 2025. Lecture Notes in Computer Science. Cham: Springer, 15725. 10.1007/978-3-031-95911-0_12

[B25] Mehdipour Ghazi M. Urdanibia-Centelles O. Bakhtiari A. Fagerlund B. Vestergaard M. B. Larsson H. B. W. (2025). Cognitive aging and reserve factors in the Metropolit 1953 Danish male cohort. GeroScience 47, 2475–2493. 10.1007/s11357-024-01427-2 39570569 PMC11978600

[B26] Mihai P. G. Tschentscher N. Von Kriegstein K. (2021). Modulation of the primary auditory thalamus when recognizing speech with background noise. J. Neurosci. 41, 7136–7147. 10.1523/JNEUROSCI.2902-20.2021 34244362 PMC8372015

[B27] Nielsen J. B. Dau T. (2011). The Danish hearing in noise test. Int. J. Audiol. 50, 202–208. 10.3109/14992027.2010.524254 21319937

[B28] Nilsson M. Soli S. D. Sullivan J. A. (1994). Development of the Hearing In Noise Test (HINT) for the measurement of speech reception thresholds in quiet and in noise. J. Acoust. Soc. America. 95, 1085–1099. 10.1121/1.408469 8132902

[B29] Nuesse T. Steenken R. Neher T. Holube I. (2018). Exploring the link between cognitive abilities and speech recognition in the elderly under different listening conditions. Front. Psychol. 9, 678. 10.3389/fpsyg.2018.00678 29867654 PMC5968383

[B30] Osler M. Andersen A. M. N. Lund R. Batty G. D. Hougaard C. Ø. Damsgaard M. T. (2004). Revitalising the Metropolit 1953 Danish male birth cohort: background, aims and design. Paediatr. Perinat. Epidemiol. 18, 385–394. 10.1111/j.1365-3016.2004.00584.x 15367326

[B31] O’Brien J. L. Lister J. J. Fausto B. A. Morgan D. G. Maeda H. Andel R. (2020). Are auditory processing and cognitive performance assessments overlapping or distinct? Parsing the auditory behaviour of older adults. Int. J. Audiol. 60, 1–10. 10.1080/14992027.2020.1791366 32701036

[B32] Rönnberg J. Hygge S. Keidser G. Rudner M. (2014). The effect of functional hearing loss and age on long- and short-term visuospatial memory: evidence from the UK biobank resource. Front. Aging Neurosci. 6, 1–13. 10.3389/fnagi.2014.00326 25538617 PMC4260513

[B33] Sanchez-Lopez R. Sørensen C. B. Pedersen D. L. Pedersen C. Schmidt J. H. Laugesen S. (2026). Evaluating the user-operated audible contrast threshold test as a language-independent proxy of speech-in-noise perception. Int. J. Audiol. 22, 1–8. 10.1080/14992027.2026.2622388 41724196

[B34] Slade K. Plack C. J. Nuttall H. E. (2020). The effects of age-related hearing loss on the brain and cognitive function. Trends Neurosci. 43, 810–821. 10.1016/j.tins.2020.07.005 32826080

[B35] Soli S. D. Wong L. L. N. Assessment of speech intelligibility in noise with the hearing in noise test. Int. J. Audiology vol. 47 356–361. 10.1080/14992020801895136 2008). 18569108

[B36] Svobodová V. Profant O. Škoch A. Tintěra J. Tóthová D. Chovanec M. (2024). The effect of aging, hearing loss, and tinnitus on white matter in the human auditory system revealed with fixel-based analysis. Front. Aging Neurosci. 15, 1283660. 10.3389/fnagi 38264549 PMC10803717

[B37] Wang Y. R. Bacon B. A. Champoux F. Théoret H. (2026). Pure tone auditory thresholds and their association with cognition in the Canadian longitudinal study on aging. Sci. Rep. 16 (1), 5808. 10.1038/s41598-026-36979-0 41559162 PMC12894998

[B38] Windle R. Dillon H. Heinrich A. (2023). A review of auditory processing and cognitive change during normal ageing, and the implications for setting hearing aids for older adults. Front. Neurol. 14, 1–17. 10.3389/fneur.2023.1122420 PMC10318159 37409017

[B39] World Health Organization (2021). World Report on Hearing. Human Rights Watch. Geneva, Switzerland: World Health Organization.

[B40] Wu M. Christiansen S. Fereczkowski M. Neher T. (2022). Revisiting auditory profiling: can cognitive factors improve the prediction of aided speech-in-noise outcome? Trends Hearing 26, 23312165221113889. 10.1177/23312165221113889 PMC9373127 35942807

[B41] Zaar J. Simonsen L. B. Sanchez-Lopez R. Laugesen S. (2024). The audible contrast threshold (ACT) test: a clinical spectro-temporal modulation detection test. Hear. Res. 453, 109103. 10.1016/j.heares.2024.109103 39243488

